# Inhibition of AMPA receptor trafficking at hippocampal synapses by β-amyloid oligomers: the mitochondrial contribution

**DOI:** 10.1186/1756-6606-3-10

**Published:** 2010-03-26

**Authors:** Yanfang Rui, Jiaping Gu, Kuai Yu, H Criss Hartzell, James Q Zheng

**Affiliations:** 1 Departments of Cell Biology and Neurology, Center for Neurodegenerative Diseases, Emory University School of Medicine, Atlanta, GA 30322, USA

## Abstract

**Background:**

Synaptic defects represent a major mechanism underlying altered brain functions of patients suffering Alzheimer's disease (AD) [1-3]. An increasing body of work indicates that the oligomeric forms of β-amyloid (Aβ) molecules exert profound inhibition on synaptic functions and can cause a significant loss of neurotransmitter receptors from the postsynaptic surface, but the underlying mechanisms remain poorly understood. In this study, we investigated a potential contribution of mitochondria to Aβ inhibition of AMPA receptor (AMPAR) trafficking.

**Results:**

We found that a brief exposure of hippocampal neurons to Aβ oligomers not only led to marked removal of AMPARs from postsynaptic surface but also impaired rapid AMPAR insertion during chemically-induced synaptic potentiation. We also found that Aβ oligomers exerted acute impairment of fast mitochondrial transport, as well as mitochondrial translocation into dendritic spines in response to repetitive membrane depolarization. Quantitative analyses at the single spine level showed a positive correlation between spine-mitochondria association and the surface accumulation of AMPARs. In particular, we found that spines associated with mitochondria tended to be more resistant to Aβ inhibition on AMPAR trafficking. Finally, we showed that inhibition of GSK3β alleviated Aβ impairment of mitochondrial transport, and effectively abolished Aβ-induced AMPAR loss and inhibition of AMPAR insertion at spines during cLTP.

**Conclusions:**

Our findings indicate that mitochondrial association with dendritic spines may play an important role in supporting AMPAR presence on or trafficking to the postsynaptic membrane. Aβ disruption of mitochondrial trafficking could contribute to AMPAR removal and trafficking defects leading to synaptic inhibition.

## Background

Alzheimer's disease (AD) often attacks aged populations and is highlighted by progressive loss of memory and cognitive abilities [4]. AD brains exhibit two major pathological hallmarks: extracellular senile plaques containing β-amyloid aggregates and intracellular neurofibrillary tangles consisting of hyperphosphorylated microtubule-associated *tau *proteins [5,6]. β-amyloid (Aβ) molecules are generated by proteolytic cleavage of the transmembrane β-amyloid precursor protein (APP) [7,8]. Aggregated Aβ fibrils constitute the core of neuritic plaques and are believed to be a major culprit for neurodegeneration and subsequent cognitive abnormalities in AD patients [9-11]. Recent studies, however, indicate that Aβ molecules exert adverse effects on neuronal functions independent of cell death. Specifically, soluble Aβ oligomers were found to exert severe inhibition of synaptic functions and plasticity [1,12-14], including impairment of long-term potentiation (LTP) and facilitation of long-term depression (LTD) of central synapses [15,16]. Therefore, a better understanding of Aβ inhibition of synaptic functions would provide significant insights into the AD neuropathogenic process, potentially leading to better strategies for prevention and treatment of AD.

A major mechanism to modify synaptic strength is to alter the number, types, or properties of neurotransmitter receptors at the postsynaptic terminal [17-20]. The major ionotropic glutamate receptors involved in excitatory synaptic transmission are alpha-amino-3-hydroxy-5-methyl-4-isoxazolepropionic acid receptors (AMPARs) and *N*-methyl *D*-aspartate receptors (NMDARs). AMPARs are best studied for their rapid trafficking into and out of the synapse by cycling between intracellular stores and the cell surface during synaptic potentiation and depression, respectively [19-22]. NMDARs, due to their voltage-dependent blockade by Mg^2+^, are thought to function as a coincidence detector of presynaptic and postsynaptic firing and act as the trigger of LTP. It has been shown that activity-dependent trafficking of NMDARs also plays an important role in synaptic plasticity and its alteration may contribute to neuropsychiatric disorders [23]. There is an increasing body of evidence to show that Aβ molecules, especially soluble Aβ oligomers, exert a negative impact on glutamate receptor trafficking in central synapses, leading to synaptic deficits. For example, soluble Aβ oligomers have been shown to bind to AMPARs [24] or NMDARs [25] to cause their internalization, leading to inhibition of LTP and synaptic activity. However, the precise cellular mechanisms underlying Aβ effects on glutamate receptors remain to be elucidated.

Mitochondria are a vital organelle involved in many, if not all, functions of cells. Not only are mitochondria the main energy source of the cell, but they also serve as a part of intracellular Ca^2+ ^stores and regulate intracellular Ca^2+ ^homeostasis, and most importantly regulate cell apoptosis [26-29]. Mitochondria are mostly produced in the cell body and transported to specific cellular locations of increased energy needs such as synapses. It is clear that synaptic transmission and remodeling require localized mitochondria to generate ATP as well as to control local Ca^2+ ^concentrations [30,31]. While mitochondria are known to accumulate at the presynaptic terminal for neurotransmitter release[32], localization of mitochondria to the postsynaptic terminals has also been demonstrated [33]. Our previous study showed that soluble Aβ molecules acutely impair mitochondrial movement in cultured hippocampal neurons [34]. We thus speculated that disruption of mitochondrial localization to synapses may exert adverse effects on synaptic functions. In this study, we utilized live-cell imaging to investigate whether soluble Aβ oligomers adversely affect AMPAR trafficking at the postsynaptic terminal and its potential mitochondrial connection. We show that soluble Aβ oligomers caused acute reduction of AMPARs on the spine surface and impaired AMPAR insertion during chemically-induced LTP. Furthermore, Aβ oligomers rapidly impaired mitochondrial transport and translocation into dendritic spines. Our analyses revealed that mitochondrial localization to spines is positively correlated to the presence/insertion of AMPARs on the spine surface. Finally, inhibition of GSK3β prevented Aβ inhibition of both mitochondrial transport and AMPAR trafficking. Together, these findings indicate that mitochondrial localization to dendritic spines may be important for AMPAR trafficking and acute Aβ impairment of mitochondrial trafficking could contribute to the adverse effects of Aβ on AMPARs at synapse.

## Results

### Aβ oligomers decrease surface AMPARs and inhibit TEA induced surface AMPAR increase at dendritic spines

**Figure 1:**
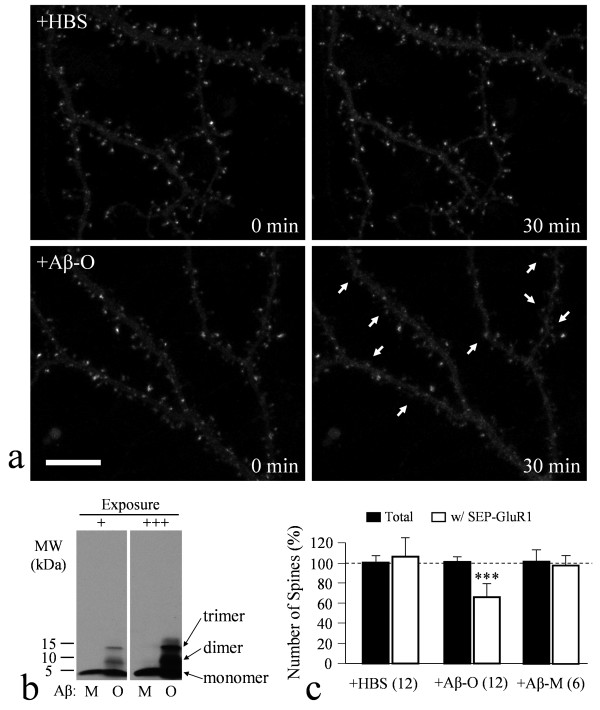
**Aβ-induced loss of surface AMPARs as revealed by confocal live-cell imaging of SEP-GluR1**. (**a**) Representative images of dendritic regions of cultured hippocampal neurons (DIV21) expressing SEP-GluR1 before and after 30 min exposure to the control saline (HBS, upper panels) or 5 μM Aβ_1-42 _solution (lower panels). SEP-GluR1 signals are mostly seen concentrating in dendritic spines. Arrows indicate the spines exhibiting substantial loss of SEP-GluR1 signals after 30 min exposure to Aβ. Scale bar: 10 μm. (**b**) Western blots showing the existence of Aβ monomers and oligomers in our Aβ preparation. Two different exposures (+ short, +++ long) were shown to ensure that no additional Aβ aggregates in Aβ-M and Aβ-O preparations. (**c**) Quantitative analysis showing the number of total spines or spines exhibiting strong SEP-GluR1 fluorescence after 30 min exposure to HBS or Aβ. The data were normalized against the number before the 30 min exposure with 100% indicating no change. Triple asterisks: p < 0.0005 comparing to the corresponding control group (Student's *t*-test).

We took advantage of pH-dependent fluorescence emission of pHluorin molecules and expressed a super-ecliptic pHluorin fused to the N-terminus of the AMPAR glutamate receptor 1 (SEP-GluR1) in cultured hippocampal neurons for live-cell imaging of AMPAR trafficking. The strong fluorescence of SEP at pH ≥ 7.0 (e.g. extracellular solution: pH 7.4) allowed us to detect the surface presence and dynamic changes of SEP-GluR1 at the single spine level [35,36]. We found that SEP-GluR1-expressing neurons exhibited numerous spines along the dendritic processes, many of which exhibited strong SEP-GluR1 fluorescence (Figure 1a, also see Figure S1 in Additional file 1). On the other hand, the dendritic shaft, as well as a substantial number of the spines, displayed a low level of diffuse SEP-GluR1 fluorescence, which was considered as the background signals of unclustered SEP-GluR1. These imaging data are consistent with the notion that spines represent the postsynaptic terminals of excitatory synapses with concentrated glutamate receptors. We confirmed that the strong SEP-GluR1 fluorescence came from surface SEP-GluR1 as it was effectively quenched by cell-impermeable acidic buffer (Additional file 1, Figure S1).

Recent studies have shown that long term exposure to Aβ oligomers decreases synaptic AMPAR number and impairs AMPAR trafficking [24,37]. We therefore tested if Aβ oligomers exert any acute effects on surface AMPARs. We prepared an oligomeric Aβ solution (Aβ-O, see Methods) and western blotting confirmed the presence of dimers and trimers, as well as monomers (Figure 1b). Based on the western blot, the amount of oligomers (dimers and trimers) is less than 10% of the monomers in this Aβ preparation. We found that a 30 min exposure of hippocampal neurons to 5 μM Aβ-O (<500 nM oligomers, based on the western blot) resulted in a marked loss of surface SEP-GluR1 at numerous spines (lower panels in Figure 1a, arrows; see also Figure S2 in Additional file 1), whereas similar exposure to a control saline (HBS) had no effect (upper panels in Figure 1a; see also Figure S2 in Additional file 1). We quantified the numbers of spines exhibiting SEP-GluR1 fluorescence before and after 30 min treatment using an intensity threshold that cut off the baseline fluorescence of dendritic shaft (see Methods and Figure S2 in Additional file 1). Our results show that Aβ-O resulted in ~40% reduction in the number of spines emitting strong SEP-GluR1 fluorescence, while the total number of spines was not changed (Figure 1c). To test if Aβ oligomers were the ones affecting AMPARs, we prepared an Aβ solution containing only monomers (Aβ-M; Figure 1b). Our data showed that Aβ monomers had no effect on SEP-GluR1 signals at dendritic spines (Figure 1c), confirming the notion that Aβ monomers do not affect neuronal viability and functions [1,38]. Together, our data show that Aβ oligomers in submicromolar concentration markedly reduce surface AMPARs at postsynaptic terminals.

**Figure 2:**
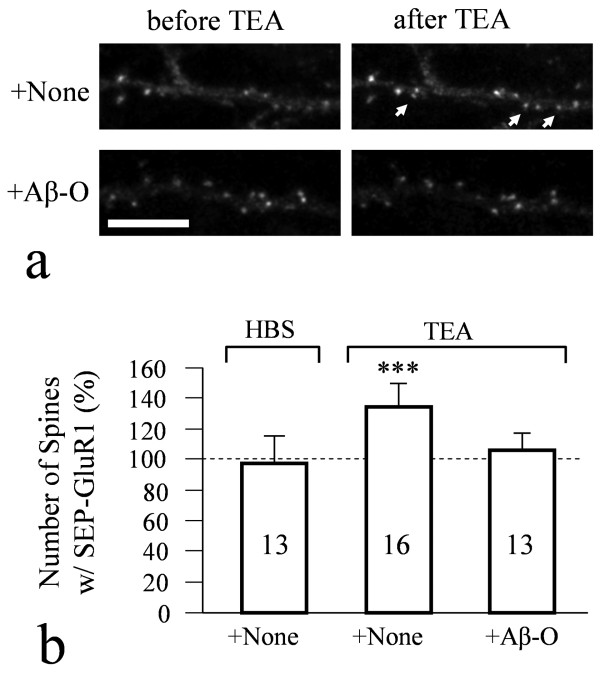
**Aβ inhibition of AMPAR insertion during chemical LTP**. (**a**) Representative images showing the increase of SEP-GluR1 elicited by 10 min TEA treatment with (bottom pair) and without (top pair) 30 min exposure to Aβ oligomers. (**b**) Quantification of the number of spines exhibiting strong SEP-GluR1 fluorescence after 10 min exposure to either HBS (control) or 25 mM TEA with and without Aβ treatment. The data were normalized against the number before 10 min exposure to either HBS or TEA with 100% indicating no change. Triple asterisks: p < 0.0005 comparing to the HBS control (Student's *t*-test).

To study the influence of Aβ oligomers on AMPAR trafficking during synaptic plasticity, we adopted a method to chemically induce LTP (cLTP) by a brief exposure of cells to a potassium channel blocker tetraethylammonium (TEA), which has been shown to robustly elicit AMPAR-dependent LTP in brain slices [39]. We exposed mature (DIV21) hippocampal neurons expressing SEP-GluR1 to 25 mM TEA in a high-calcium and low-magnesium environment for 10 min and examined changes in SEP-GluR1 fluorescence at spines. The potentiation of synaptic efficacy by this cLTP method was confirmed by whole cell patch-clamp recording of both spontaneous and miniature excitatory postsynaptic currents (Additional file 1, Figure S3). We found that a 10 min TEA exposure resulted in a marked increase in the number of spines exhibiting strong SEP-GluR1 fluorescence (Figure 2a, arrows; see also Figure S1 in Additional file 1). Quantification of the number of spines exhibiting bright SEP-GluR1 fluorescence before and after cLTP showed about 30% increase, whereas the control saline did not cause any changes (Figure 2b). Pre-treatment of neurons with 5 μM Aβ-O for 30 min eliminated TEA-induced increase in spines exhibiting bright SEP-GluR1 fluorescence (Figure 2b). Therefore, Aβ oligomers inhibited AMPAR insertion during synaptic potentiation.

### Aβ oligomers acutely inhibit mitochondria trafficking

**Figure 3:**
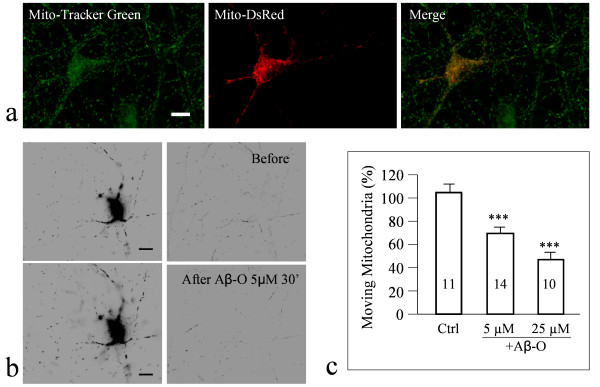
**Acute impairment of mitochondrial movement by Aβ oligomers as revealed by time-lapse imaging of Mito-DsRed expressing neurons**. (**a**) Representative images of a hippocampal neuron expressing Mito-DsRed (red), which was also stained by MitoTracker (green). The merged image (the 3^rd ^panel) shows that Mito-DsRed and MitoTracker signals are perfectly colocalized in this transfected neuron, whereas MitoTracker also labeled many more mitochondria in surrounding cells. (**b**) Representative images from time-lapse sequences before and after 30 min exposure to Aβ_1-42 _oligomers. The left panels represent a snapshot of the mitochondrial distribution in a Mito-DsRed-expressing neuron. The right panels represent the movement traces of mitochondria derived from 5-min time-lapse sequences. The movement traces were generated from the time-lapse sequence using Zprojection followed by division against the first frame using ImageJ software [34]. (**c**) Quantitative analysis showing the number of moving mitochondria after 30 min treatment with the control saline and different concentration of Aβ-O. The data were normalized against the number of moving mitochondria before the 30 min treatment with 100% indicating no change. Triple asterisks: p < 0.0005 (comparing to vehicle, Student's *t*-test).

**Figure 4:**
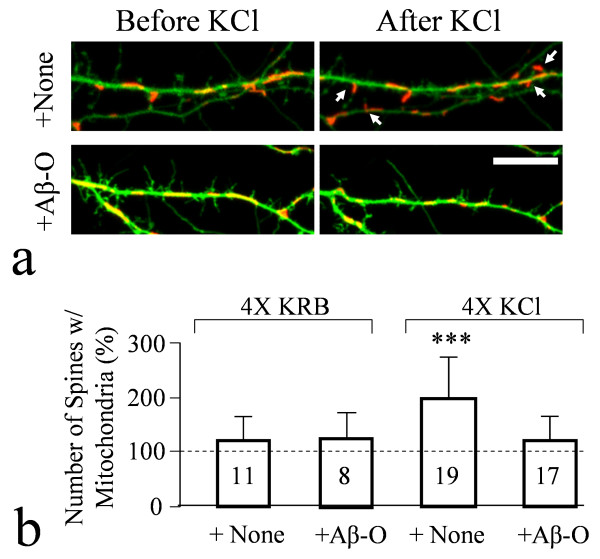
**Aβ impairment of mitochondrial trafficking into spines in response to repetitive KCl depolarization**. (**a**) Representative images of dendritic segments of hippocampal neurons expressing GFP and Mito-DsRed before and after repetitive KCl depolarization (4X), with (lower pair) and without (upper pair) 30 min exposure to Aβ oligomers. Arrows indicate spines containing mitochondria after the repetitive KCl depolarization. Scale bar: 10 μm. (**b**) Quantification of the number of spines containing mitochondria after repetitive exposure (4 times) to either KRB (control) or KCl, with and without Aβ treatment. The data are normalized against the number of spines before the repetitive exposure with 100% indicating no change. Triple asterisks: p < 0.0005 compared to the control (Student's *t*-test).

Many mitochondria in neurons display microtubule-dependent fast movement in both axonal and dendritic processes, which could be acutely impaired by soluble Aβ molecules [34,40]. Here we further confirmed that the oligomeric form of Aβ exerted acute impairment of fast mitochondrial movement in hippocampal neurons (Figure 3), whereas Aβ monomers had no effect (Additional file 1, Figure S4). Mitochondria also translocate into dendritic protrusions (filopodia and spines) in response to neuronal activity, which may play a role in synapse development and plasticity [33]. We thus examined if Aβ oligomers also inhibit activity-dependent mitochondrial translocation into dendritic spines. Consistently, we found that repetitive KCl depolarization caused a significant increase in the number of spines containing mitochondria (Figure 4a). Quantitative analysis showed that the number of spines containing mitochondria almost doubled after repetitive KCl treatment (Figure 4b). However, 30 min exposure of the neurons to 5 μM Aβ-O completely blocked the increase of mitochondrial translocation into spines by repetitive KCl depolarization (Figure 4b). Repetitive exposure of neurons to the control saline (KRB) with and without Aβ-O in bath did not affect the number of spines containing mitochondria with and without Aβ presence. Therefore, Aβ oligomers appear to impair fast mitochondrial movement as well as trafficking to postsynaptic terminals in response to membrane depolarization.

### Aβ inhibition of surface AMPAR trafficking: a potential mitochondrial contribution

**Figure 5:**
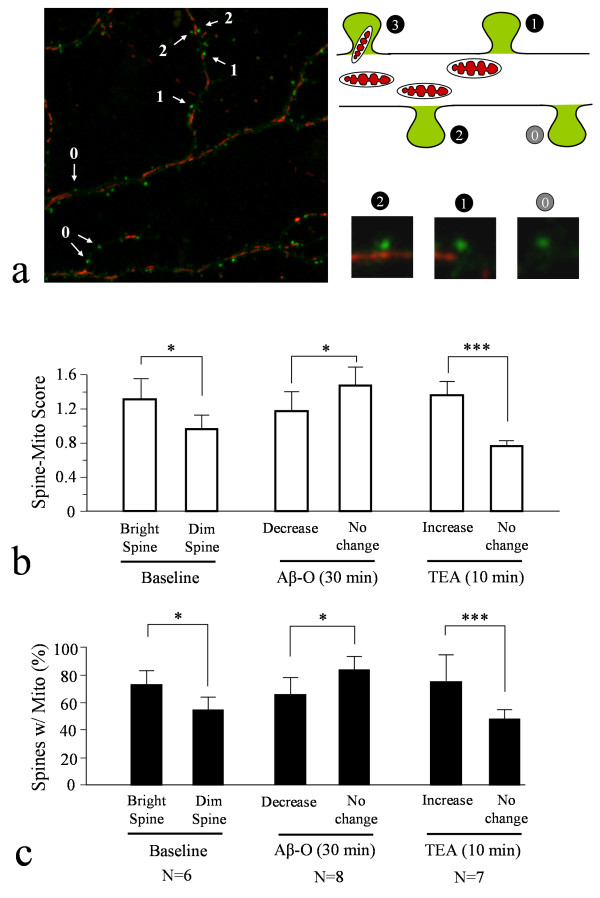
**Spine-mitochondria association and its contribution to AMPAR trafficking**. (**a**) A scoring system to assign different spine-mito scores to each spine. The color image on the left shows a merged image of SEP-GluR1 highlighted spines (green) and mitochondria (red). Different scores (0-3) were given to spines depending on their association and proximity to a mitochondrion. The schematic diagram illustrates the scoring criteria, together with examples in magnified view. Analysis of several hundreds of spines from at least three batches of experiments was performed for each condition and data are summarized in the bar graph in (**b**). Spines receiving spine-mito scores of 1-3 were considered to be associated with mitochondria and used for calculating the percentage of spines associated with mitochondria (**c**). Single asterisk: p < 0.05; double asterisk: p < 0.005; triple asterisks: p < 0.0005 (comparing to the corresponding control group, Student's *t*-test).

Given that Aβ oligomers similarly inhibited trafficking of both AMPARs and mitochondria, we hypothesized that a close localization/association of mitochondria to spines may be important for the maintenance of AMPARs on the spine surface, as well as for their increase during synaptic potentiation. To test this hypothesis, we performed extensive analysis on the association of mitochondria with spines exhibiting strong SEP-GluR1 fluorescence or ones with weak fluorescence similar to that of dendritic shaft. We hereafter referred to these two types of spines as *bright *and *dim *spines, respectively, for our analysis. Here, hippocampal neurons expressing both SEP-GluR1 and Mito-mOrange were examined by live-cell confocal imaging. Merged color images of SEP-GluR1 and Mito-mOrange show that many spines, especially those bright spines, have mitochondria positioned nearby (Figure 5a). To analyze spine-mitochondria association, we developed a scoring system to give each spine a spine-mito score (Figure 5a): 3 = mitochondria inside the spine, 2 = mitochondria in dendritic shaft but spanning across the entire spine base, 1 = mitochondria in dendritic shaft only partially covering the spine base or immediately adjacent to the spine, 0 = no mitochondria in the vicinity of at least two-spine distance. Our analysis of several hundreds spines showed that, at baseline, bright spines had a significant higher spine-mito score than dim spines (Figure 5b). We also determined the percentage of spines associated with mitochondria (spines with spine-mito scores of 1-3) and found that, consistently, more bright spines were associated with mitochondria than dim spines (Figure 5c). These results suggest that mitochondrial association with spines may favor surface presence of AMPARs in spines.

Using the same analysis, we next examined the loss of surface AMPARs induced by acute Aβ-O exposure. Both the spine-mito score and the percentage of spines associated with mitochondria showed a significant difference between bright spines that lost SEP-GluR1 signals and those exhibited no change under Aβ-O exposure (Figure 5b &5c). Generally, spines exhibiting SEP-GluR1 loss had a lower spine-mito score and less mitochondrial association than those without loss. We next performed similar analysis on the potential association of mitochondria with spines exhibiting AMPAR insertion during cLTP. We found that dim spines exhibiting marked increase in SEP-GluR1 fluorescence had a higher spine-mito score and more mitochondrial association than those without SEP-GluR1 increase (Figure 5b &5c). Taken together, these data suggest that spines associated with mitochondria tend to favor AMPAR insertion during cLTP and appear to be more resistant to AMPAR loss induced by Aβ oligomers.

**Figure 6:**
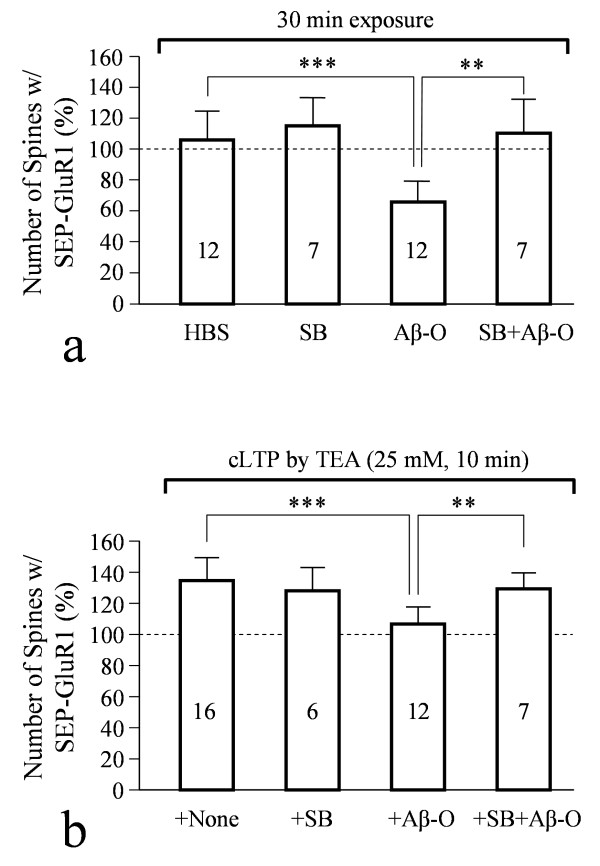
**Involvement of GSK3β in Aβ impairment of AMPAR trafficking**. Inhibition of GSK3β by a specific inhibitor SB415286 abolished both Aβ-induced loss of AMPARs from spine surface (**a**) and Aβ-inhibition of AMPAR insertion during chemical LTP (**b**) as revealed by SEP-GluR1 imaging. Double asterisks: p < 0.005; triple asterisks: p < 0.0005 (comparing to the control, Student's *t*-test).

Glycogen synthase kinase-3β (GSK3β) is known to play an important role in Aβ toxicity [41-43] and our previous study showed that inhibition of GSK3β alleviated Aβ impairment of mitochondrial transport [34]. We hence tested if GSK3β inhibition could also mitigate Aβ inhibition of AMPAR trafficking. Using a specific GSK3β inhibitor SB415286 [44,45], we found that both Aβ-induced loss of AMPARs and inhibition of AMPAR insertion during cLTP were largely abolished (Figure 6). These data thus suggest a potentially shared pathway for Aβ impairment of mitochondrial transport and AMPAR trafficking. Taken together, our results suggest that mitochondrial trafficking and localization to spines may be important for the maintenance of surface AMPARs at spines at the resting state and their increase during plasticity. Acute impairment of mitochondrial trafficking by Aβ oligomers could potentially contribute to or accelerate Aβ-induced loss of surface AMPAR and inhibition of AMPAR insertion during synaptic potentiation.

## Discussion

Soluble Aβ oligomers have been shown to impair synaptic functions but the underlying mechanisms remain to be fully understood. At the postsynaptic side of excitatory synapses, Aβ-induced internalization of neurotransmitter receptors has been considered to contribute to reduced synaptic strength, but how Aβ oligomers reduce surface receptors is unclear. In this study, we used live-cell imaging to investigate the acute effects of soluble Aβ molecules on AMPAR trafficking at the postsynaptic terminal and the potential contribution of mitochondria. This study was partially inspired by our previous findings that soluble Aβ molecules acutely inhibit mitochondrial movement in hippocampal neurons, independent of cell death and other drastic alternations of cellular structures [34]. Given that mitochondria are a crucial organelle for energy supply and intracellular Ca^2+ ^regulation, impaired mitochondrial movement could disrupt their proper localization to synaptic sites, thus contributing to synaptic deficits elicited by Aβ molecules. Taking advantage of the pH-dependent fluorescence emission of SEP-GluR1, we were able to quantitatively analyze surface AMPARs, their trafficking during cLTP, and the effects of Aβ oligomers at single spine level. Such an imaging-based approach has allowed us to perform detailed analysis of changes associated with individual spine. For instance, we were able to show that Aβ-induced removal of surface AMPARs was not a consequence of spine loss, thus supporting a relatively direct action of Aβ on AMPAR trafficking [24]. Furthermore, when combined with mitochondrial imaging, we were able to reveal a positive correlation between spine localization of mitochondria and AMAPR trafficking. It is quite intriguing to see that local presence of mitochondria appears to favor AMPAR insertion during synaptic potentiation and make them less prone to Aβ inhibition.

While our findings on Aβ-induced removal of surface AMPARs and inhibition of insertion during synaptic potentiation were based on imaging of exogenously expressed SEP-GluR1, we have performed surface staining using an anti-GluR1 antibody and confirmed the live imaging results (unpublished results). Furthermore, our results are consistent with previous studies employing electrophysiology, immunostaining, and live-cell imaging in which Aβ was shown to reduce surface AMPARs [24,37,46]. Aβ-induced reduction of surface AMPARs has been shown to share a common pathway with long term depression (LTD) and to involve Ca^2+ ^signaling through calcineurin for clathrin-mediated endocytosis of AMPARs [24]. On the other hand, how Aβ inhibits AMPAR insertion during cLTP is unclear. Given that AMPAR insertion during LTP depends on Ca^2+^-dependent exocytosis, Aβ-elicited LTD pathway and elevated AMPAR endocytosis could jeopardize LTP signaling cascades to impair AMPAR insertion. While we considered the increase in SEP-GluR1 fluorescence after TEA-cLTP a result of increased AMPAR insertion, our data could not rule out the possibility of decreased AMPAR internalization by TEA. Nonetheless, our study here has provided an intriguing possibility that Aβ impairment of mitochondrial trafficking might contribute to Aβ inhibition on AMPARs. Localization of mitochondria to both pre- and post-synaptic terminals has been observed and likely plays a crucial role for synaptic transmission and remodeling [31-33,47,48]. The rapid inhibition of mitochondrial movement observed previously [34,40] could potentially disrupt the synaptic localization of mitochondria to adversely affect synaptic functions. Indeed we found that a brief exposure of hippocampal neurons to Aβ oligomers inhibited mitochondrial translocation into spines induced by repetitive membrane depolarization. Based on our correlation analysis, the lack of mitochondrial association appears to facilitate the inhibition of AMPAR trafficking by Aβ oligomers.

How do mitochondria contribute to AMPAR trafficking? Potentially, the local production of ATP by mitochondria is required for vesicular fusion and insertion of AMPARs to the postsynaptic surface. Mitochondria could also be involved in local regulation of intracellular Ca^2+ ^concentrations that are crucial for numerous synaptic activities including synaptic transmission, LTP and LTD, and endo/exocytotic trafficking of membrane proteins. In particular, both LTP and LTD depend on Ca^2+ ^signaling to control synaptic receptor trafficking: the former requires a high Ca^2+ ^elevation for activating CaMKII and downstream effectors for AMPA insertion whereas the latter needs small Ca^2+ ^signals to activate calcineurin phosphatase for AMAPR removal from the surface [20,49,50]. The lack of mitochondria at the postsynaptic terminal could alter local Ca^2+ ^signals to favor the LTD pathway for AMPAR removal [24], thus impeding the LTP-induced AMPAR insertion. Certainly, many other synaptic activities, such as ATP-driven ion pumps and local protein synthesis could also depend on the local presence of mitochondria, which could be disrupted by Aβ oligomers. While Aβ disruption of mitochondrial trafficking and localization to synapses might not directly or solely cause AMPAR trafficking defects, it could significantly contribute to postsynaptic defects in coordination and synergy with other Aβ-elicited events (e.g. Aβ induced internalization of synaptic receptors). While direct evaluation of this mitochondrial hypothesis requires selective disruption of mitochondrial localization to spines or of specific mitochondrial function(s) at spines, our findings that inhibition of GSK3β mitigate Aβ impairment of trafficking of both AMPAR and mitochondria suggest that these two events could be linked in contributing to Aβ-induced synaptic inhibition.

In conclusion, our studies showed that soluble Aβ oligomers exert acute inhibition on the trafficking of both mitochondria and synaptic receptors. The postsynaptically localized mitochondria appear to be important for the maintenance of AMPARs on postsynaptic surface as well as for AMPAR insertion during synaptic potentiation. Intriguingly, our correlation analysis suggests that impairment of mitochondrial trafficking might contribute to the adverse effects of Aβ oligomers on AMPARs on the postsynaptic surface. Future studies that employ selective targeting of mitochondrial movement could provide more definite answers regarding the precise role of mitochondria in synaptic receptor trafficking, as well as its precise contribution to synaptic defects in AD brains.

## Methods

### Cell culture and transfection

Hippocampal neurons from embryonic day 18 rats were obtained according to the method described previously [51]. Dissociated cells were plated in 35 mm glass bottom culture dishes (Warner Instruments, Hamden, CT) for culture and microscopy. The glass surface was pretreated with 100 μg/ml poly-D-lysine (Sigma, St. Louis, MO) overnight and ~200,000 cells were plated in each dish in Neurobasal medium containing B27 and Glutamax (Invitrogen). Cells were maintained in a 5% CO_2 _incubator at 37°C, with half of the culture medium replaced with fresh Neurobasal medium every 3 d. Before each imaging experiment, the medium was replaced by Krebs'-Ringer's buffer (KRB, in mM: 150 NaCl, 5 KCl, 2 CaCl_2_, 1 MgCl_2_, 10 glucose, and 10 HEPES, pH 7.4) [52] or HEPES-buffered solution (HBS, in mM: 140 NaCl, 5 KCl, 2 CaCl_2_, 1.5 MgCl_2_, 10 glucose, and 25 HEPES, pH 7.4).

Hippocampal neurons were transfected using CalPhos Mammalian Transfection Kit (Clontech, Mountain View, CA). Neurons plated in 35 mm culture dishes at different days in vitro (DIV) were used depending on the experiments. Typically, we transfected the cells several days before the imaging experiments to allow the expression of various GFP-fusion or mutant proteins. For experiments on mitochondrial transport, we typically transfected the neurons at DIV6-7 and performed imaging on DIV8-9. For KCl depolarization experiments, the transfection was performed on DIV12-13 and followed by imaging on DIV14-15. For imaging studies on AMPARs, the transfection was performed on DIV13-14 followed by imaging on DIV21-22 when mature synaptic connections had been formed. The DNA constructs for transfection were prepared by plasmid maxi kit (Qiagen, Valencia, CA). The following constructs were used: Mito-DsRed and Mito-GFP (generously provided by Dr. Zheng Li at NIH/NIMH), pCi-SEP-GluR1 (a gift from Dr. Roberto Malinow at University of California at San Diego), EGFP-C1 and mOrange (Clontech). To create Mito-mOrange, the mOrange coding sequence was subcloned into Mito-GFP vector with green fluorescent protein (GFP) sequence excised.

### Aβ preparation and treatment

We followed the previously published method to prepare Aβ oligomers for our experiments (Aβ-O solution) [53]. Aβ_1-42 _was purchased from American Peptide Company Inc (Sunnydale, CA) and dissolved in hexafluoro-2-propanol (HFIP) and aliquoted to microfuge tubes. HFIP was subsequently removed by evaporation in a speed-vacuum and desiccated Aβ aliquots were stored at -20°C. To make Aβ oligomer solution, each Aβ_1-42 _aliquot was dissolved in DMSO to make a 5 mM stock solution. The solution was diluted to 100 μM with KRB and kept at 4°C for 24 hr before use. To make an Aβ solution containing only monomers (Aβ-M solution), Aβ_1-42 _was directly dissolved in ddH2O at 1 mM, diluted to 100 μM with KRB, and incubated at 37°C for 7 d. Afterwards, the Aβ solution was centrifuged at 14,000 rpm for 60 min to remove Aβ fibrils. The supernatant was collected and passed through a 100 KD molecular weight cut-off (MWCO) Amicon centrifugal filter (Millipore) to further remove any large Aβ aggregates. Western blotting showed that this method produced only Aβ monomers (Figure 1b). The concentration of Aβ monomers in solution was determined using Bradford Protein Assay (Bio-Rad) and adjusted to the same concentration of Aβ-O solution.

Bath application of Aβ was achieved through a two-step dilution procedure. First, the Aβ stock solution was diluted in KRB to twice the designated concentration (2× working stock). The 2× working stock solution was then gently added to and mixed with the bath saline of the cells in an equal volume to reach the desired final concentration. In a typical experiment, 1 ml of the 2× stock solution was added to 1 ml of the bath solution in the culture/imaging dish on the microscope stage.

### Western blotting to detect Aβ molecules

We used 4G8 anti-Aβ antibody (Signet, Dedham, MA) to perform western blotting to detect different forms of Aβ in our preparation. 80 ng Aβ samples were added to sample buffer with 50 mM DTT and heated at 85°C for 2 min. Samples were loaded and fractioned by PAGE on 10-20% Tris-Tricine gel (Invitrogen) and subsequently transferred to nitrocellulose membranes. The membrane was boiled for 10 min in PBS and blocked with 5% non-fat dry milk in TBS with 0.05% Tween-20 (TBST) for 1 h at room temperature. The membrane was then incubated with 4G8 antibody (1:1000) in blocking buffer overnight at 4°C. Bound antibodies were detected by HRP-conjugated secondary antibody, visualized by chemiluminescence using ECL (Thermo Scientific, Rockford, IL), and quantified using the gel analysis routine of ImageJ software (NIH).

### Live cell imaging of mitochondrial movement

Fluorescent time-lapse recordings were performed on an inverted microscope (TE2000, Nikon) using a 40× N.A. 1.3 S-Fluor oil immersion objective with identical settings between the control and experimental groups. Time-lapse images were captured with a CCD camera (SensiCam QE, Cooke Scientific) using the IPLab imaging software (BD Biosciences). For imaging of mitochondrial transport, we typically recorded neurons at a sampling rate of one frame every 5 s for 5 min, with the CCD exposure at 50 ms exposure and 2 × 2 binning. For each experiment, a population of neurons was imaged for a 5 min control period before the application of Aβ molecules, followed by another 5 min time-lapse recording at 30 min after Aβ application. All the experiments were performed on the microscope stage with the 35 mm dish housed in a temperature controlled chamber (Warner Instruments, New Haven, CT) with the temperature set at ~35°C. Quantification of moving mitochondria was done by simply counting the number of moving mitochondria in each 5 min time-lapse sequence. A moving mitochondrion was defined as one that moved more than a distance of twice its length over the 5 min period. Since no change in the total mitochondrial number was observed [34], we normalized the number of moving mitochondria in the 5-min sequence against that before the Aβ application. A value of 100% indicates that same numbers of moving mitochondria were observed in both recording periods.

### Confocal live-cell imaging on mitochondrial association with dendritic spines and AMPAR trafficking

A Nikon C1 confocal on TE300 inverted microscope, together with a 60× N.A.1.4 Plan Apo oil immersion objective, was used for imaging. To be able to examine all the spines at different focusing planes of a dendritic segment, a z-stack of 10-12 images was taken on a selected dendritic region followed by maximal intensity projection to generate the 2-D image. For experiments on KCl-stimulated mitochondria translocation into spines, two-channel confocal imaging was performed on neurons expressing EGFP and Mito-DsRed at DIV14-15. To stimulate mitochondrial translocation into spines, we used a previously described method of repetitive membrane depolarization by KCl [33]. Here, 90 mM NaCl of normal KRB was replaced with 90 mM KCl (hereafter referred to as KCl-KRB) for membrane depolarization. We performed 4 times of KCl-KRB exposure, each exposure for 3 min and separated by 10 min recovery in normal KRB. The same neurons were imaged before and one hour after the 4× KCl stimulation to examine the association of mitochondria with spines.

Similar confocal imaging was performed on hippocampal neurons expressing SEP-GluR1 to study AMPAR trafficking. Since SEP-GluR1 only emits strong fluorescence on cell surface and forms clusters as endogenous AMPARs at postsynaptic terminals, we used an intensity threshold that cut off the diffuse SEP-GluR1 fluorescence of dendritic shaft (considered as background) to select postsynaptic receptor clusters that emitted substantial SEP-GluR1 signals, followed by quantification of their number. Both thresholding and quantification were done using ImageJ software. To examine the effect of Aβ molecules on surface AMPAR clusters, we acquired images of the same dendritic region before and after Aβ exposure, followed by same thresholding and quantification to determine the change in the number of spines with SEP-GluR1 signals. For AMPAR insertion during synaptic potentiation, we used a method involving a brief exposure of cells to a potassium channel blocker tetraethylammonium (TEA) to chemically induce potentiation (cLTP) [39]. Here, we stimulated mature (DIV21) hippocampal neurons expressing SEP-GluR1 with 25 mM TEA in a high-calcium and low-magnesium solution (in mM: 140 NaCl, 5 KCl, 5 CaCl_2_, 0.1 MgCl_2_, 10 glucose, and 25 HEPES, 25 TEA, pH 7.4) for 10 min. Confocal live-cell imaging on the same dendritic regions was performed before and after the stimulation to detect changes in SEP-GluR1 fluorescence. Similar thresholding and quantification were done on the two images (before and after cLTP induction) to quantify the change in the number of spines with SEP-GluR1 signals. For Aβ effects on AMPAR insertion during cLTP, we pre-treated the neurons with Aβ oligomers for 30 min before the cLTP induction by TEA.

### Electrophysiology

Conventional whole cell patch-clamp recordings were performed on the cell body of pyramidal hippocampal neurons with voltage-clamped at -70 mV using an EPC-7 patch-clamp amplifier (HEKA Instruments Inc., Bellmore, NY). Fire-polished borosilicate glass patch pipettes had a resistance of 3-5 MΩ. Experiments were conducted at room temperature (20-24°C). Since the liquid junction potentials were small (< 2 mV), no correction was made. The standard pipette solution contained (mM): 147 KCl, 2 KH_2_PO_4_, 5 Tris-HCl, 2 EGTA, 10 HEPES, 4 Mg-ATP, pH 7.3 adjusted with KOH, and osmolarity at 310-320 mOsmol^-1^. The extracellular recording solution contained (mM):128 NaCl, 5 KCl, 2 CaCl_2_, 1 MgCl_2_, 25 HEPES, 30 glucose, 0.1 picrotoxin, pH 7.3 with NaOH, and osmolarity at 300-310 mOsmol^-1^. For miniature EPSCs, 0.5 μM tetrodotoxin (TTX) was added to the extracellular recording solution. To induce synaptic potentiation, a TEA solution (in mM: 80 NaCl, 20 KCl, 2 CaCl_2_, 25 TEA, 25 HEPES, 30 glucose, pH7.3 and 315 mOsmol^-1^) was perfused to the neurons. We typically recorded for 5-10 min before and after 10 min TEA treatment (25 mM). During the TEA treatment, the patch-clamp amplifier was switched to the current clamp mode with the current set to zero for maximal synaptic stimulation. The cell was re-clamped at -70 mV after TEA washout. Recorded EPSCs were filtered at 2 kHz before the analysis and presentation.

## Competing interests

The authors declare that they have no competing interests.

## Authors' contributions

YR performed a majority of the experiments and analyses, and wrote the first draft of the manuscript. JG helped with live imaging and analysis. KY of Hartzell lab did the electrophysiological recordings and HCH provided feedback on the manuscript. JQZ designed, planned, guided the project, as well as did some imaging experiments and writing. All authors have read and approved the final manuscript.

## Supplementary Material

Additional file 1 **The additional file**1**contains supplemental figures S1-S4.** Click here for file
