# Supplementary material for: Inhibition of AMPA receptor trafficking at hippocampal synapses by β-amyloid oligomers: the mitochondrial contribution
Source: Mol Brain. 2010 Mar 26;3:10. doi: 10.1186/1756-6606-3-10 (PMC2853530; doi:10.1186/1756-6606-3-10)
Supplement: Additional file 1 — The additional file1contains supplemental figures S1-S4. [file 1756-6606-3-10-S1.PDF]

Additional file 1 – Rui et al.

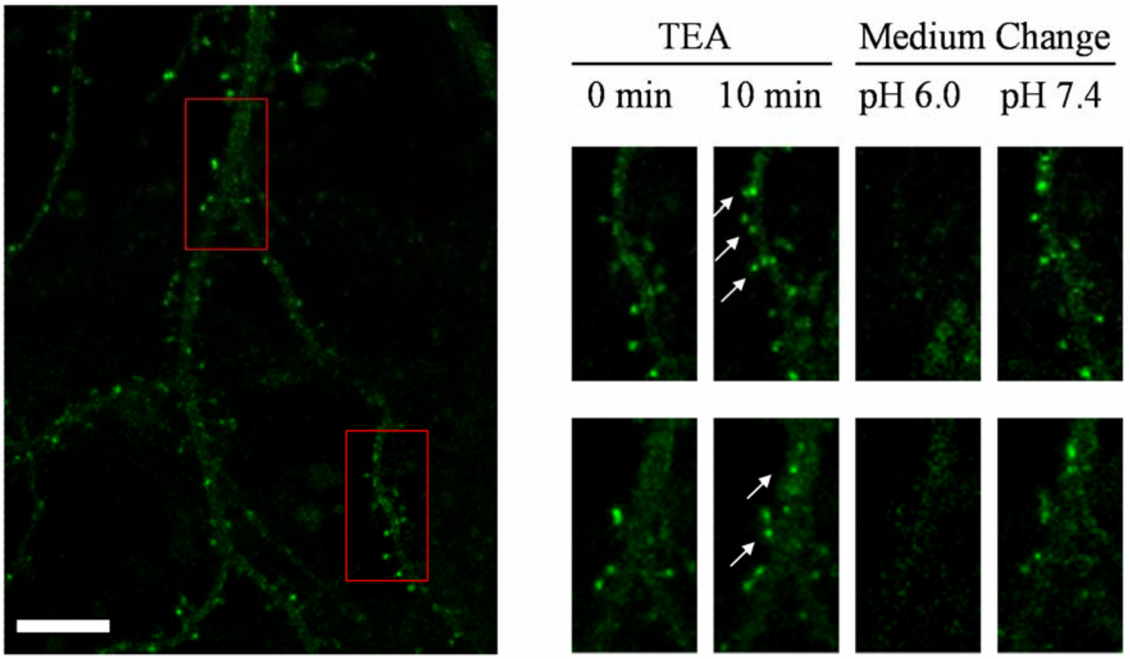

Figure S1. Live confocal imaging of SEP-GluR1 as an effective approach to detect surface AMPARs and their trafficking during chemical LTP. Left large panel: a representative image showing a dendritic region of a DIV21 hippocampal neuron expressing SEP-GluR1. Many spines are highlighted by strong SEP-GluR1 fluorescence. Right panels: two small dendritic regions (indicated by the red rectangles on the left panel) are shown in magnified view before and after 10 min cLTP induction by 25 mM TEA treatment. Arrows: new insertion. The SEP-GluR1 fluorescence was effectively quenched by an acidic buffer (in mM: 140 NaCl, 5 KCl, 2 $CaCl_2$, 1.5 $MgCl_2$, 10 glucose, and 25 MES, and pH 6.0) and restored by a buffer with pH7.4, indicating that it represented surface SEP-GluR1.

Additional file 1 – Rui et al.

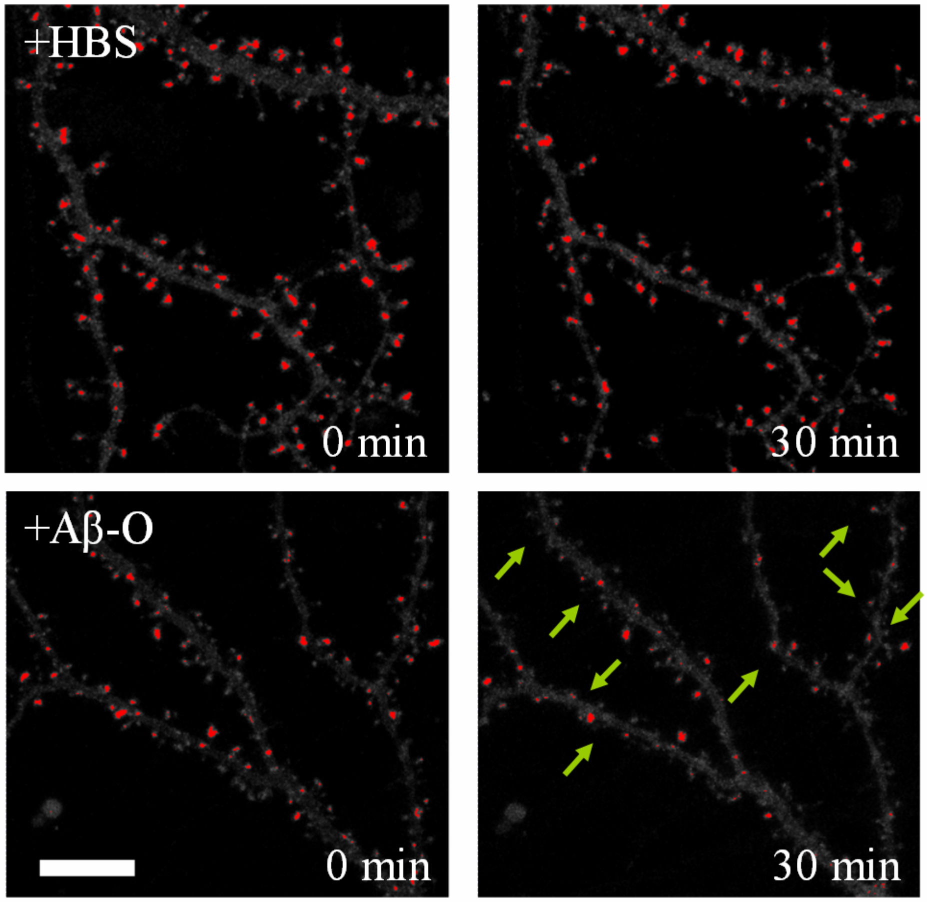

Figure S2. Representative color images of dendritic regions of cultured hippocampal neurons (DIV21) expressing SEP-GluR1 before and after 30 min exposure to the control saline (HBS, upper panels) or 5 μM Aβ-O solution (lower panels). These images are the same ones shown in Figure 1a, but with bright spines highlighted in red after the application of an intensity threshold that cut off the baseline fluorescence of the dendritic shaft. Arrows indicate the spines exhibiting substantial loss of SEP-GluR1 signals after 30 min exposure to Aβ. Scale bar: 10 μm.

Additional file 1 – Rui et al.

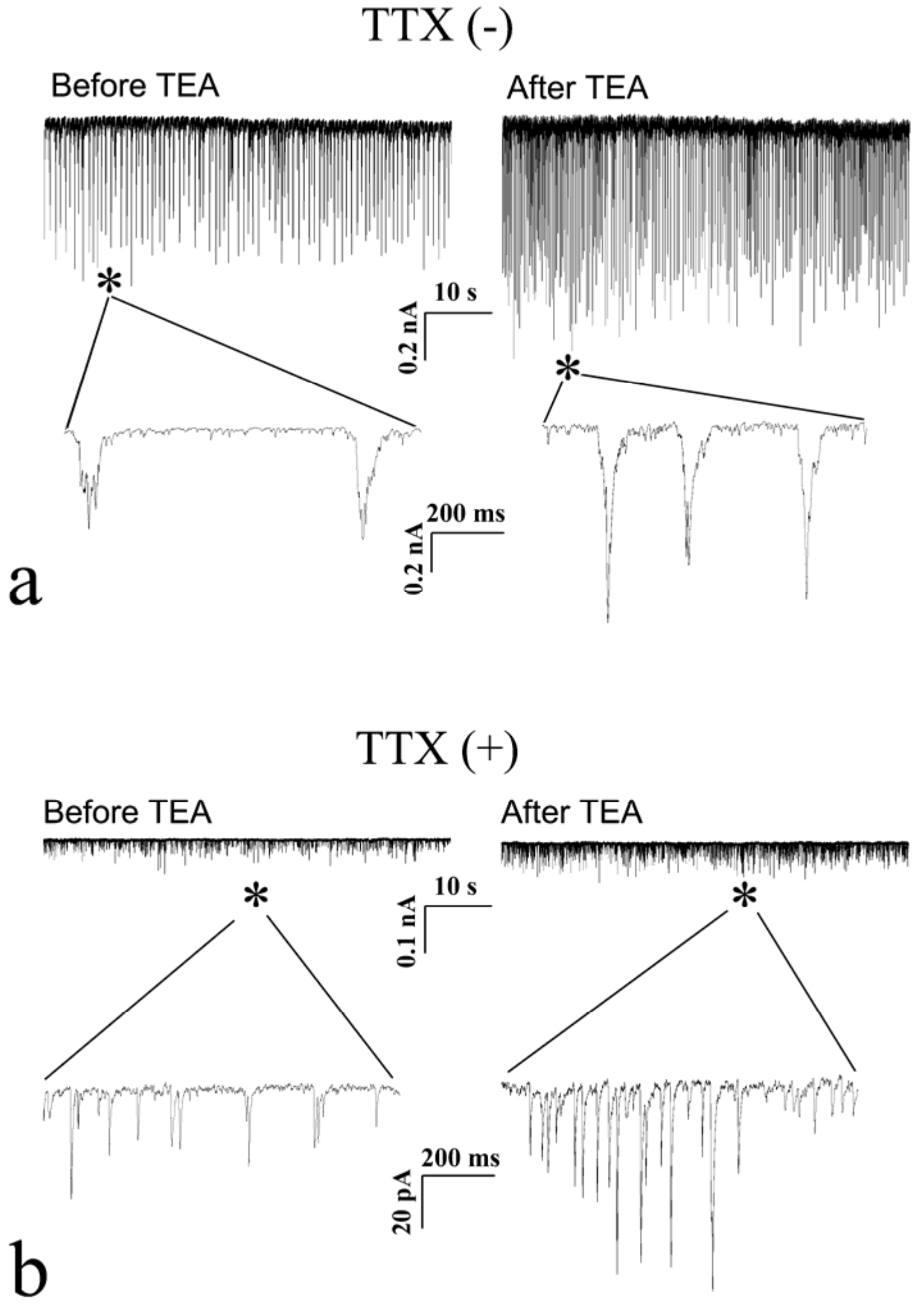

Figure S3. Whole cell patch-clamp recordings of spontaneous (a) and miniature (b) excitatory postsynaptic currents (EPSCs) before and after 10 min exposure to 25 mM TEA. Representative sEPSCs and mEPSCs from DIV21 hippocampal neurons are shown. Recordings were performed with the cell voltage clamped at -70 mV. 10 min TEA treatment was done in the absence of voltage clamp and TTX.

Additional file 1 – Rui et al.

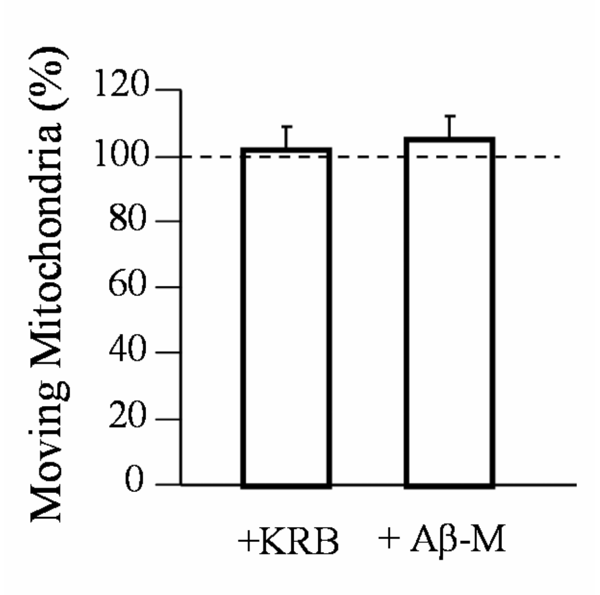

Figure S4. No effect of Aβ monomers on mitochondrial movement. Mitochondria were labeled by vital dye MitoTracker Green and time-lapse recordings were performed before and after 30 min exposure to the control saline (KRB) or Aβ-M (~ similar concentration to 5 µM Aβ-O). The number of moving mitochondria after 30 min exposure to KRB or Aβ-M was normalized against that before the exposure. A value of 100% indicates no change in the number of moving mitochondria.
